# Novel breast cancer screening: combined expression of miR-21 and MMP-1 in urinary exosomes detects 95% of breast cancer without metastasis

**DOI:** 10.1038/s41598-019-50084-5

**Published:** 2019-09-19

**Authors:** Wataru Ando, Kiyoshi Kikuchi, Takayuki Uematsu, Hiroaki Yokomori, Takashi Takaki, Masaya Sogabe, Yutaka Kohgo, Katsuya Otori, Shigemi Ishikawa, Isao Okazaki

**Affiliations:** 1 Department of Clinical Pharmacy, Center for Clinical Pharmacy and Sciences, Kitasato University School of Pharmacy, 5-9-1 Shirokane, Minato-ku, Tokyo 108-8641 Japan; 2 Department of Surgery, Sanno Hospital, International University of Health and Welfare, 8-10-16 Akasaka, Minato-ku, Tokyo 107-0052 Japan; 3 Biomedical Laboratory, Division of Biomedical Research, Kitasato University Medical Center, 6-100 Arai, Kitamoto City, Saitama 364-8501 Japan; 4 Department of Internal Medicine, Kitasato University Medical Center, 6-100 Arai, Kitamoto City, Saitama 364-8501 Japan; 5 Division of Electron microscopy, Showa University School of Medicine, 1-5-8 Hatanodai, Shinagawa-ku, Tokyo 142-8555 Japan; 6 Department of General Thoracic Surgery, Jichi Medical University, 3311-1 Yakushiji, Shimotsuke, Tochigi 329-0498 Japan; 7 Department of Internal Medicine, International University of Health and Welfare Hospital, 537-3 Iguchi, Nasu-Shiobara, Tochigi 329-2763 Japan; 8 Health Care Center, International University of Health and Welfare Hospital, 537-3 Iguchi, Nasu-Shiobara, Tochigi 329-2763 Japan; 9 Department of Chest Surgery, International University of Health and Welfare Hospital, 537-3 Iguchi, Nasu-Shiobara, Tochigi 329-2763 Japan; 10 Department of Internal Medicine, Sanno Hospital, International University of Health and Welfare, 8-10-16 Akasaka, Minato-ku, Tokyo 107-0052 Japan

**Keywords:** Breast cancer, Predictive markers

## Abstract

Serum and tissue miR-21 expression in patients with breast cancer (BC) is a useful biomarker for cancer diagnosis, progression, and treatment. Matrix metalloproteinase-1 (MMP-1) is also important in breast cancer carcinogenesis. However, miR-21 and MMP-1/CD63 in urine exosomes in these patients have not been examined. Urine samples were collected from patients with BC and 26 healthy females. Urinary exosomes were isolated and confirmed by western blotting with anti-CD63 antibody and electron microscopy observation. MiR-21 and MMP-1/CD63 expression was examined by quantitative RT-PCR and western blotting, respectively. Patients with very early stage breast cancer were evaluated. MiR-21 expression in the patients was 0.26 [95% CI: 0.20–0.78], which was significant lower than in the 26 controls (1.00 [95% CI: 1.01–3.37], p = 0.0947). MMP-1/CD63 expression in patients was significantly higher than in controls (1.74 [95% CI: 0.86–5.08] vs 0.535 [95% CI: −0.01–2.81], p = 0.0001). Sensitivity and specificity were 0.708 and 0.783 for miR-21 and 0.792 and 0.840 for MMP-1/CD63, respectively. Sensitivity and specificity of combined expression were 95% and 79%, respectively. The sensitivity of MMP-1/CD63 expression in urinary exosomes was better than that of miR-21 expression. Thus, miR-21 and MMP/CD63 may be useful markers for BC screening.

## Introduction

Breast cancer (BC) is prevalent worldwide. In the United States, the incidence of BC was high in the 1990s, but has somewhat decreased^1^. In Japan, however, the prevalence of BC has gradually increased, with the highest incidence rate and the third leading cause of mortality among all cancers^2^. Early detection of BC is associated with a good prognosis, and thus BC screening has been widely promoted for the past 30 years in Japan. However, mammography and/or echography is time-consuming and costly, as well as inconvenient.

We previously reported urinary cancer screening tests for measuring urinary 3-hdroxyproline^3–5^. However, the reliability of these tests was low. Screening methods for BC that are simple, reliable, easy to access, and inexpensive are needed. Molecular biology studies showed that exosomes secreted from cancer cells can migrate to distant organs to form niches, leading to metastasis^6,7^. Exosomes contain both microRNAs (miRs) and oncogenes^6,7^. The expression levels of miR-21 in the tissue and serum derived from patients with BC have been reported as useful biomarkers for diagnosis, cancer progression, and treatment selection^8–16^. MiR-21 inhibits the function of several tumour suppressor genes^17,18^. The expression of miR-21 in urinary exosomes of patients with BC has not been reported because they had not confirmed their samples by CD63 antibody nor by the electron microscopy observation^16^.

We also previously investigated the mechanism of matrix metalloproteinase-1 (MMP-1) in the formation of liver cirrhosis^19–23^ and development of hepatocellular carcinoma^24–26^. MMP-1 is a key proteinase in matrix metabolism^27^. MMP-1 not only attacks collagen molecules at the three quarters from N-terminals^27^ but also is involved in cancer development including BC^27–29^.

This study was performed to determine the expression levels of both miR-21 and MMP-1/CD63 in urinary exosomes and evaluate these biomarkers for screening of early stage BC.

## Results

### Clinical characteristics and histopathological findings of 22 patients with BC

The clinical characteristics and histopathological findings of the 22 patients with BC are shown in Table 1. Cases were not advanced at surgical resection; one case showed metastasis at 6 months after surgical resection. These cases were detected relatively early because most patients visited the Department of Preventive Medicine of Sanno Medical Center for a health check which included BC screening, and suspicious lesions were detected by breast echography and mammography. Two cases were under 40 years old, 11 cases were between 40 and 49 years old, 5 cases were between 50 and 59 years old, one case was 61 years old, and 3 cases were 72, 74, and 74 years old. Therefore, 16 cases (72%) were between 40 and 59 years old. Among the 22 patients, two patients were in Stage 0, 7 patients were in Stage I, 7 patients were in Stage II, and the remaining 6 patients were in Stage III. Regarding tumour size, 11 cases were 1–2 cm, 6 cases were 2–5 cm, and 5 cases were >5 cm. Seven cases were positive in the lymph nodes. Ten cases showed scirrhous carcinoma, 3 cases showed mucinous carcinoma, 2 cases showed solid tubular carcinoma, 2 cases showed papillary tubular carcinoma, 2 cases showed invasive lobular carcinoma, and 3 cases were microinvasion, invasive ductular carcinoma, and ductular carcinoma *in situ*.

**Table 1:** Breast Cancer Cases, TNM Classification, and Characteristics.

No.	Age (years)	Body Weight (kg)	BMI (kg/m^2^)	TNM classification*	Stage	Patho^†^	Expression Levels of miR-21 (2^ΔCt^)^#^	Expression Levels of MMP-1/CD63^##^
1	61	110	40.4	T2N0M0	II	scirrhous ca.	0.11	1.78
2	59	53	19.5	TisN0M0	0	microinvasive-	0.26	1.02
3	36	67	24.3	T2N0M0	II	solid tub. ca.	0.09	1.70
4	56	49	19.1	T1N0M0	II	scirrhous ca.	1.04	2.00
5	46	53	21.5	T2N0M0	I	solid tub. ca.	0.37	1.08
6	40	68	26.9	T3N1M0	IIIA	pap-tub.ca.	0.18	0.76
7	52	52	20.8	T1N1M0	IIA	scirrhous ca.	0.32	1.09
8	52	63	26.1	T2N1M0	IIB	scirrhous ca.	0.22	0.85
9	48	54	20.4	T1N0M0	I	scirrhous ca.	0.30	1.84
10	74	69	25.3	T2N0M0	II	mucinous ca.	0.08	3.04
11	49	56	22.4	T1N0M0	I	scirrhous ca.	0.00	23.20
12	48	62	22.5	T1N0M0	I	pap-tub. ca.	0.06	3.56
13	46	65	22.8	T1 N0 M0	I	ductal ca. *in situ*	0.01	2.25
14	47	54	20.1	T3 N3 M0	IIIC	scirrhous ca	0.01	3.91
15	47	70	27.0	T1 N0 M0	I	scirrhous ca	0.36	2.23
16	45	57	22.7	Tis N0 M0	0	inv lob ca	0.26	6.78
17	74	57	24.3	T3 N0 M0	II	scirrhous ca	0.23	3.90
18	52	50	16.7	T3N0M0	IIIA	inv. lobular ca	0.49	1.53
19	72	61	26.8	T3N1M0	IIIA	mucinous ca	0.41	0.78
20	45	50	19.8	T1cN3M0	IIIA	inv. ductal ca	2.27	1.02
21	46	52	20.6	T1N3M0	IIIA	scirrhous ca	1.48	0.92
22	35	58	20.1	T2N0M0	I	mucinous ca	2.22	0.11
						Median (95% CI)	0.26 (0.20–0.73)	1.74 (0.86–5.08)

*TNM classification; ^†^Pathological diagnosis; ca: carcinoma; tub: tubular; pap: papillary.

^#^Number indicates the relative expression levels, that is microRNA copies by RT-PCR in patients divided the mean number of microRNA copies in healthy controls.

^##^Number indicates the relative expression levels of MMP-1/CD63, that was measured by western blotting with both antibodies.

### Isolation and confirmation of urinary exosomes

**Figure 1:**
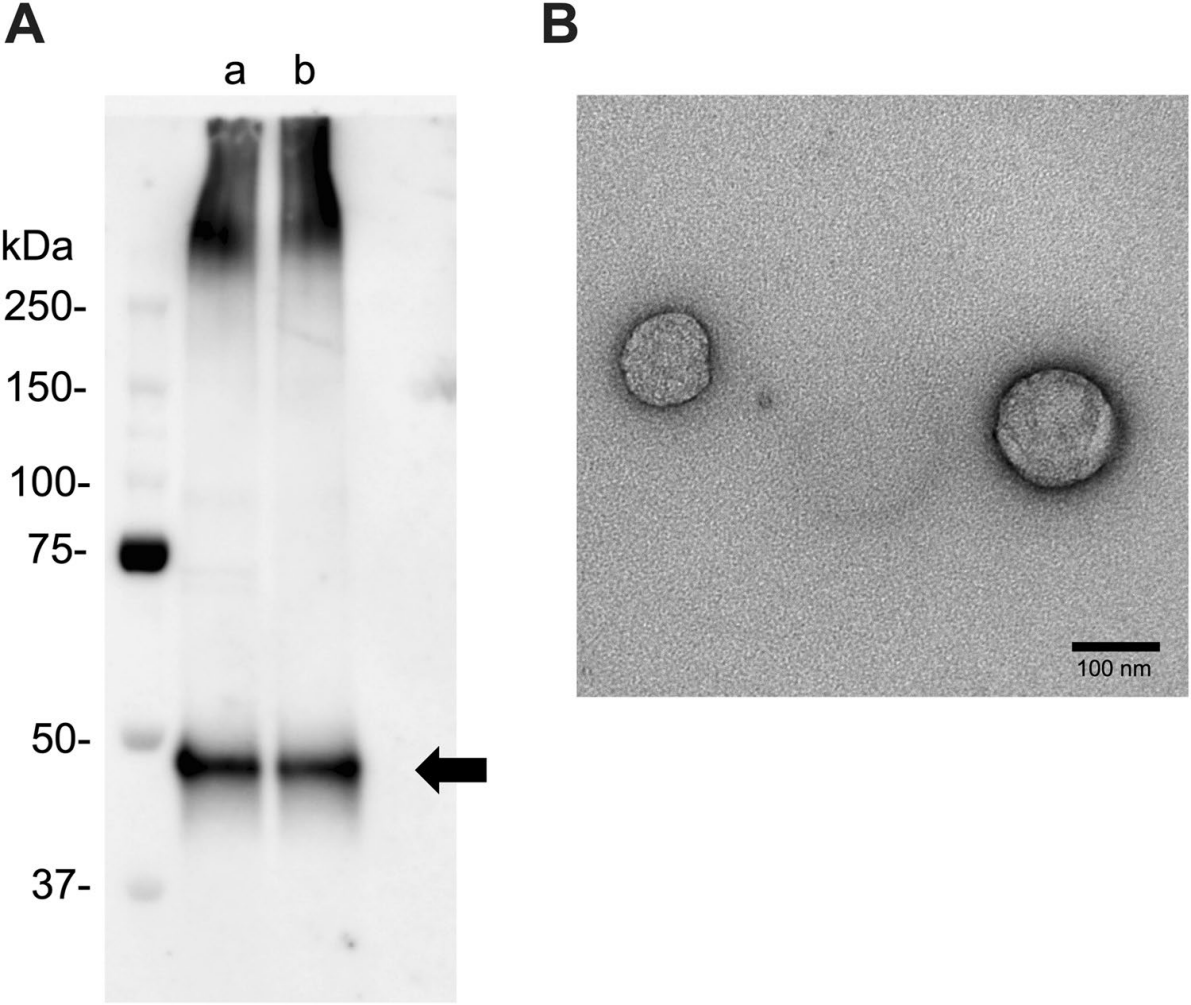
(**A**) Western blot analysis of urinary exosome in patients with BC and healthy controls. (a) Patients with BC (No. 1) and (b) Healthy control (No. 1). One-sixth of the urinary exosome extracted from 2 mL urine was applied to the wells. anti-CD63 antibody biotin conjugated (1:1000). The blot is full-length and no trimming. (**B**) Electron microscopy of urinary exosomes. The size of exosomes was nearly 100 nm and the EM features were identified.

Urinary exosomes isolated with a Miltenyi Biotec isolation kit (Bergisch Gladbach, Germany) were confirmed to be exosomes by western blotting with anti-CD63 antibody (Fig. 1A) and the size of isolated exosomes was determined by electron microscopy (Fig. 1B).

### Expression levels of miR-21 and MMP-1/CD63 in 22 patients with BC and 26 healthy females

**Figure 2:**
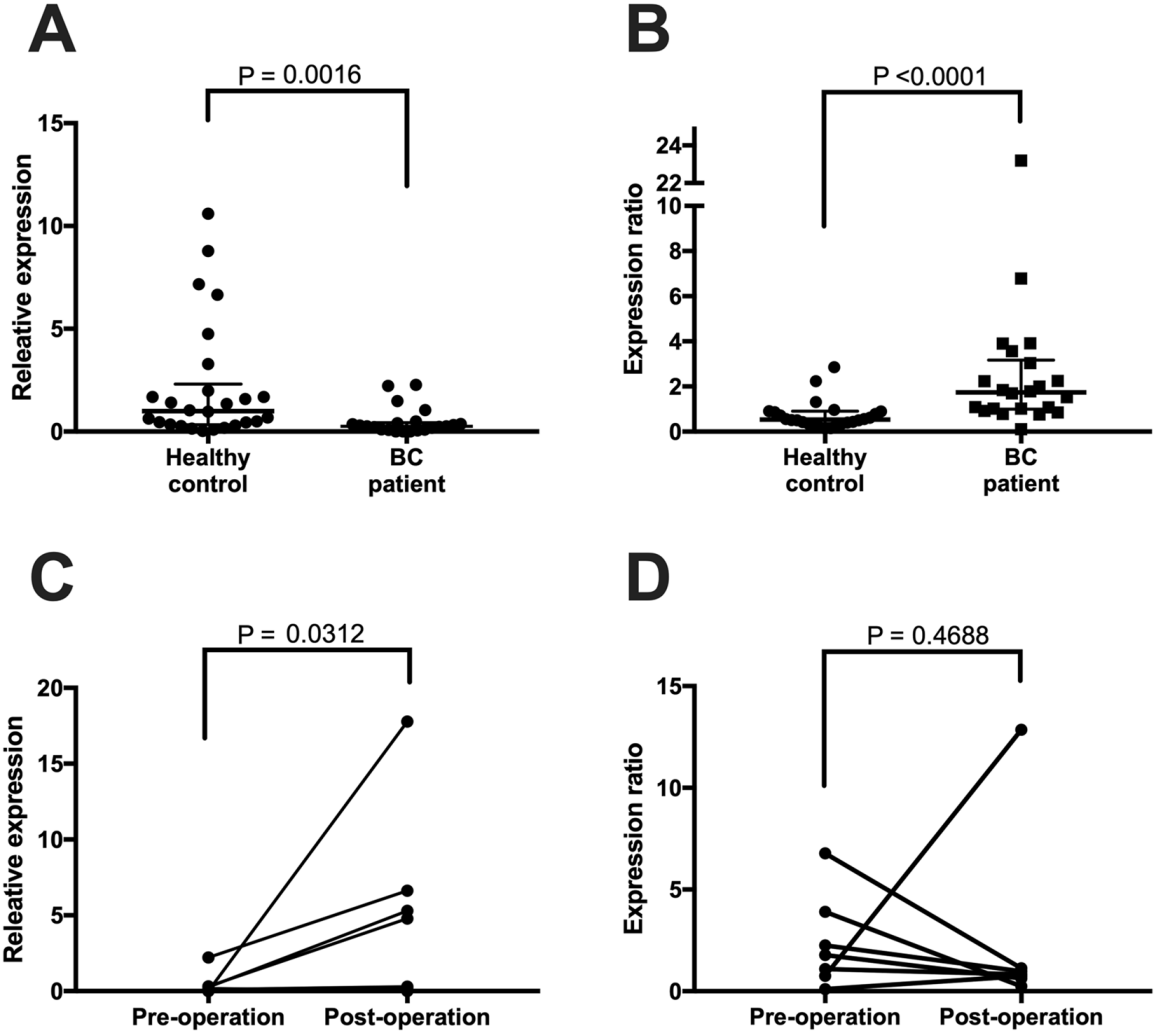
(**A**) Comparison of miR-21 expression urinary exosomes between patients with BC and healthy controls. Exosomal miR-21 expression in BC patients is shown as relative expression based on CT value of miR-21 in 26 healthy controls. BC, breast cancer; CT, threshold cycle. (**B**) Comparison of MMP-1/CD63 expression in urinary exosomes between BC patients and healthy controls. (**C**) Comparison of miR-21 expression pre- and post-operation. (**D**) Comparison of MMP-1/CD63 expression pre- and post-operation. Exosomal expression of MMP-1/CD63 was based on the ratio of the expression of western blotting.

The relative expression levels of miR-21 in 22 patients with BC based on the expression levels of healthy controls are shown in Table 1, and the individual data of 26 healthy controls are listed in Supplementary Table 2. The mean expression level of 22 patients with BC was 0.26 [95% CI: 0.20–0.78], which was significantly lower than that in the 26 healthy controls (1.00 [95% CI: 1.01–3.37], p = 0.0947), as shown in Fig. 2A. The mean expression levels of miR-21 in healthy controls in the 60–79 years group was 1.84, which was higher than that in the 30–39 years group (0.68), but the difference was not significant (Table 2).

**Table 2:** Age group of Healthy Controls and Expression Levels of miR-21 and MMP-1.

Age group (years)	Number of healthy controls	Expression Levels of miR-21 (2^ΔCt^)^*^ [Median (95% CI)]	Expression Levels of MMP-1/CD63^**^ [Median (95% CI)]
30–39	5	0.68 (0.18–1.34)	0.30 (0.14–1.31)
40–49	12	1.02 (0.27–4.75)	0.64 (0.50–2.24)
50–59	5	1.03 (0.45–8.78)	0.43 (0.29–0.96)
60–79	4	1.84 (0.09–10.6)	0.49 (0.35–0.78)
Median (95% CI)		1.00 (1.01–3.37)	0.54 (−0.01–2.81)

*Number indicates the relative expression levels, that is microRNA copies by RT-PCR in patients divided the mean number of microRNA copies in healthy controls.

**Number indicates the relative expression levels of MMP-1/CD63, that was measured by western blotting by both antibodies.

**Figure 3:**
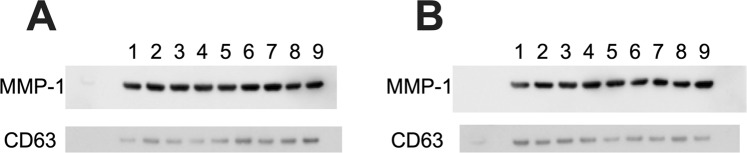
Western blot analysis of MMP-1 and CD63 in urinary exosomes in patients with BC and healthy controls. (**A**) BC patients (n = 9); No. 1–9, and (**B**) Healthy control (n = 9); No. 1–9 (shown in Table 1). One-sixth of the urinary exosome extracted from 2 mL urine was applied to the wells. Anti-MMP-1 antibody (1:1000) and anti-CD63 antibody (1:1000) were used. Samples derived from the same experiment with blots processed in parallel. Strict loading control was performed on all blots. Full-length blots are presented in Supplementary Fig. 1. BC, breast cancer.

The expression levels of MMP-1/CD63 in 22 patients are shown in Table 1 and those in the 26 healthy controls are shown in Supplementary Table 2. Figure 3 shows the process of evaluation of MMP-1/CD 63 expression by western blotting. The mean expression level in the 22 patients was 1.74 [95% CI: 0.86–5.08], which was significantly higher than that in the 26 healthy controls (0.54 [95% CI: −0.01–2.81], p = 0.0001), as shown in Fig. 2B. There was no significant difference between the 4 age groups of healthy controls in the expression levels of MMP-1/CD 63 (Table 2).

### ROC Analysis: Sensitivity and specificity of miR-21 and MMP-1/CD63 in primary screening for BC

**Figure 4:**
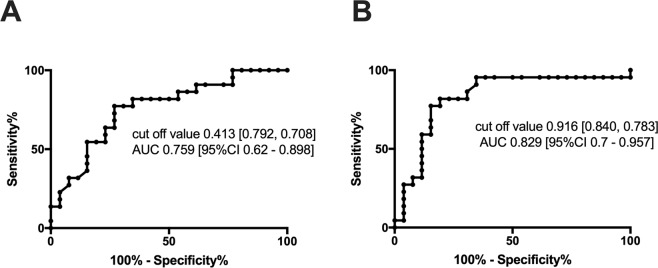
(**A**) ROC curves of miR-21 expression in urinary exosomes. When the cut-off value was 0.413, the sensitivity was 0.708 and specificity was 0.792. PPR: 0.773; NPR: 0.731. (**B**) ROC curves of MMP-1/CD 63 expression in urinary exosomes. When the cut-off value was 0.916, the sensitivity was 0.783 and specificity was 0.840. PPR: 0.818; NPR: 0.808.

The sensitivity of miR-21 in BC patients was 0.708, and the specificity of miR-21 was 0.792 in primary screening for BC against the 26 healthy controls, as shown in Fig. 4A. The sensitivity and specificity of MMP-1/CD63 expression were 0.783 and 0.840, respectively, as shown in Fig. 4B. When the expression of miR-21 and MMP-1/CD63 were combined, the final sensitivity and specificity in BC screening were 95% and 79%, respectively.

### Correlation between expression levels and each parameter of TNM classification, stage classification and histopathological findings

For miR-21, values less than the cut-off level value (0.413) were considered as positive in screening by receiver operating characteristics (ROC) analysis. For MMP-1/CD63, values higher than the cut-off level value (0.916) were considered as positive in screening. Table 3 shows the numbers of patients with positive and negative levels of miR-21 and MMP-1/CD63 in each parameter of TNM classification, stage classification, and histological findings. Using the combined expression, 95% of BC cases were detected. Table 3 shows that analysis of the expression of both miR-21 and MMP-1/CD63 detected nearly all patients with BC with Tis and T1, very small cancer, and Stage 0 and Stage I, which are very early cancers.

**Table 3:** Correlation between Expression of Both Markers and TNM Classification, Stage Classification and Characteristics in 22 Patients with Breast Cancer. (Patient Numbers).

	miR-21		MMP-1/CD63		Both	Both	Using both
	Positive	Negative	Positive	Negative	Positive	Negative	Final positive
**Tumour Size**							
Tis (2 cases)	2	0	2	0	2	0	2/2 (100%)
T1 (9 cases)	6	3	9	0	6	0	9/9 (100%)
T2 (6 cases)	5	1	4	2	4	1	5/6 (83%)
T3 (5 cases)	4	1	3	2	2	0	5/5 (100%)
							Total 21/22 (95%)
**Lymph Node Metastasis**							
N0 (15 cases)	12	3	14	1	12	1	14/15 (93%)
N1 (4 cases)	4	0	1	3	1	0	4/4 (100%)
N2 (0 cases)	0	0	0	0	0	0	0
N3 (3 cases)	1	2	3	0	1	0	3/3 (100%)
							Total 21/22 (95%)
**Stage**							
0 (2 cases)	2	0	2	0	2	0	2/2 (100%)
I (7 cases)	6	1	6	1	6	1	6/7 (93%)
II (7 cases)	6	1	6	1	5	0	7/7(100%)
III (6 cases)	3	3	4	2	1	0	6/6 (100%)
							Total 21/22 (95%)
**Pathological Findings**							
Scirrhous (10)	8	2	9	1	7	0	10/10 (100%)
Mucinous (3)	2	1	1	2	1	1	2/3 (67%)
Solid (2 cases)	2	0	2	0	2	0	2/2
Pap-tub (2)	2	0	1	1	1	0	2/2
Inv lob (2 cases)	1	1	2	0	1	0	2/2
Microinvasion (1)	1	0	1	0	0	0	1/1
Inv duct (1 case)	0	1	1	0	0	0	1/1
DCIS (1 case)	1	0	1	0	1	0	1/1
							Total 21/22 (95%)

### Changes in expression levels in seven BC cases

Seven cases provided urine samples at 20–136 weeks after surgical treatments (Table 4). Post-operative miR-21 expression was significantly increased compared to pre-operative expression (Fig. 2C). The expression levels of miR-21 were recovered to a normal range in four of seven patients after surgical intervention, whereas three cases showed still values lower than the cut-off level (0.413), as shown in Table 4 and Fig. 2C. A patient with a markedly high level of miR-21 after operation (No. 14) showed multiple bone metastasis 6 months later.

**Table 4:** Comparison of miR-21 and MMP-1/CD63 expression between pre- and post-surgical operation in seven patients with breast cancer.

No.	Pre-operation		Post-operation				
	Expression Levels of miR-21 (2^ΔCt^)^#^	Expression Levels of MMP-1/CD63^##^	Weeks since operation	Expression Levels of miR-21 (2^ΔCt^)^#^	Expression Levels of MMP-1/CD63^##^	Adjuvant chemotherapy	Metastasis/Recurrence
1	0.11	1.78	20	0.3	0.63	TC	—
6	0.18	0.76	71	0	12.86	after Trastuzumab, TC	—
7	0.32	1.09	136	4.79	0.83	Anastrozole	—
13	0.01	2.25	63	0.22	0.98	Tamoxifen	—
14	0.01	3.91	61	17.78	0.24	S-1	multiple bone metastasis
16	0.26	6.78	59	5.29	1.13	Tamoxifen	—
22	2.22	0.11	48	6.62	0.75	Tamoxifen	—
Median (95% CI)	0.18 (0.01–0.32)	4.79 (0.22–6.62)		1.78 (0.76–3.91)	0.83 (0.63–1.13)		

^#^Number indicates the relative expression levels, that is microRNA copies by RT-PCR in patients divided the mean number of microRNA copies in healthy controls.

^##^Number indicates the relative expression levels of MMP-1/CD63, that was measured by western blotting by both antibodies.

S-1, Tegafur/Gimeracil/Oteracil therapy; TC, Paclitaxel and Carboplatin therapy.

The expression levels of MMP-1/CD63 were recovered to a normal range in four of seven patients after surgical intervention, although postoperative MMP-1/CD63 expression was not significant but showed a decreasing tendency (Table 4 and Fig. 2D). Dramatically decreases were detected in four cases (No. 1, 13, 14, and 16). Three cases showed still higher levels than the cut-off level value (0.916).

Combined expression analysis revealed normal ranges in four of seven cases (Table 4). Case No. 14 showed bone metastasis with recovered levels of miR-21 and a normal range for MMP-1/CD63. Although the interaction mechanism of these markers with the clinical course including chemotherapy remains unclear, more than 50% of cases showed improved marker levels with good clinical courses after surgical treatment and chemotherapy.

### Correlation between expression levels and oestrogen receptor, progesterone receptor, and HER2

According to recent clinical classification for treatment selection, Luminal A, Luminal B, Her2, and triple-negative were analysed to detect oestrogen receptor (ER), progesterone receptor (PR), and Her2 levels (Supplementary Table 1). Table 5 shows Luminal B, Her2, and triple-negative (TN) cases were detected 100% although BC patients with Luminal A 75%.

**Table 5:** Correlation between Expression of Both Markers and Luminal A, Luminal B, Her2, and Triple Negative in 22 Patients with Breast Cancer. (Patient Numbers).

miR-21		MMP-1/CD63		both	both	using both
Positive	Negative	Positive	Negative	Positive	Negative	Final positive
**Luminal A (4 cases)**						
2	2	2	2	1	1	3/4 (75%)
**Luminal B (12 cases)**						
9	3	11	1	8	1	12/12 (100%)
**Her2 (2 cases)**						
2	0	1	1	0	0	2/2 (100%)
**Triple Negative (4 cases)**						
4	0	4	0	4	0	4/4 (100%)
						Total 21/22 (95%)

### Correlation between expression levels and clinical data in 22 patients with BC

An inverse correlation between miR-21 and MMP-1/CD63 was observed in the patients with BC (r = −0.62; p = 0.002). The correlations between both expression levels and age (less than 49 years old vs more than 50 years old), body weight, creatinine, carcinoembryonic antigen (CEA), CA15-3, Ki67, alcohol drinking habit, and smoking were analysed. A significant correlation was detected between miR-21 and body weight (r = −0.49; p = 0.02), Ki67 (r = −0.45; p = 0.02) and creatinine (r = −0.53; p = 0.01), while MMP-1/CD63 showed no correlation.

## Discussion

Several excellent screening methods for BC are available, including physical examination, mammography, ultrasonography, PET-CT, and MRI. Although these examinations are widely performed, they are costly and time-consuming. We developed a novel primary screening test based on urine collection that is inexpensive and convenient. The present study revealed that the combined expression levels of miR-21 and MMP-1/CD63 in urine exosomes can detect 95% of early BC without metastasis. However, there were some limitations. This was a pilot study performed in a single centre and few patients were available for analysis. Additional patients should be evaluated in further studies.

Detection of the expression of both miR-21 and MMP-1/CD63 in urine exosomes as a BC screening has not been reported previously. miR-21 and MMP-1 play very different biological roles, and thus their expression levels differed. miR-21 and MMP-1 are not specific to BC^27–30^. When only miR-21 is positive, other cancers (ovary, lung, liver, brain, leukemia, colorectal, pancreas, head and neck, thyroid)^30^ and liver fibrosis should be considered^31^. When only MMP-1 is positive, although lung cancer, prostate carcinoma, hepatocellular carcinoma, and other malignancies are considered^27,28^, BC should be suspected, particularly in younger women. The expression mechanism of MMP-1 in BC was recently clarified^29^. Ma *et al*.^29^ reported that miR-361-5p inhibited the proliferation of BC cells by suppressing glycolysis and inhibited BC cell invasion and metastasis by targeting MMP-1. Patients with bone disease, skin disease, and rheumatoid arthritis may also show positive results in this test.

For miR-21, the results of the present study were consistent with those of numerous previous reports of plasma or serum miR-21 without exosome isolation, which has been reported as a useful biomarker for the diagnosis, treatment selection, and future prediction of BC^8–16^. Erbes, *et al*.^16^ noted that their method was equivalent to using exosome isolation, but they did not confirm the exosomes themselves. Their results for the expression of miR-21 were consistent with those determined in this study.

The serum levels of CEA and carbohydrate antigen 153 (CA 153) have been reported as circulating biomarkers for BC. They are not useful for detecting early BC and have been used as prognostic markers for monitoring disease progression or recurrence^32^. In the present study, no cases showed higher levels of CEA and/or CA 153 (Supplementary Table 1).

Although the expression levels of miR-21 and MMP-1 in urine exosomes were not specific for BC, the present study showed good results. Ninety-five percent of BC cases were detected compared to using combined expression of both biomarkers. This percentage does not differ from those of Cohen^33^, although the number of cases in the present study was low. Further studies are necessary to confirm the usefulness of both biomarkers.

We could not conclude whether miR-21 from urine exosomes is equal to plasma or serum exosomal miR-21. MiR-21 from urine exosomes may be useful for selecting treatments after pathological diagnosis can reveal the metastasis of axillary lymph node and/or sentinel lymph node. Thus, decision making for the selection of radiation and/or chemotherapy requires miR-21 urine exosomes^34^. The present study revealed that the sensitivity for Luminal B, Her2, and TN cases was 100%, although Luminal A showed a value of 75%. A clear correlation between the percentages of ER-positive cells, PR-positive cells, and HER2-positive cells and the expression levels of miR-21 and MMP-1 were observed. Blenkiron *et al*.^35^ showed that elevated miR-21 was associated with ER-positive tumours, and Huang *et al*.^36^ reported that miR-21 was up-regulated via the MAP (ERK1/2) pathway upon stimulation of HER2 signalling in BC cells. A correlation between miR-21 and PR has not been reported^37^. These issues must be resolved to improve the selection of BC treatment, and measuring the expression of miR-21 in urinary exosomes is a convenient approach.

MiRs in the cancer exosomes inhibit the expression of their respective mRNA targets, PTEN, and HOXD10 in recipient epithelial cells^38^. Melo *et al*.^38^ showed that exosomes from BC cells transfer miRs to normal cells and stimulate them to become cancerous. Higher miR-21 expression may be related to suppression of programmed cell death 4^17^, tumour suppressor gene *TPM1*^18^, and mapin (a tumour suppressing serpin protein)^39^. Liu *et al*. reported that miR-21 induced angiogenesis through AKT and ERK activation and HIF1α expression^40^. Moreover, Fabbri et al. showed that tumour-secreted miR-21 binds to TLR8 to trigger a Toll-like receptor-mediated pro-metastatic inflammatory response that ultimately may lead to tumour growth and metastasis^41^. We observed one patient with multiple bone marrow metastasis after surgical treatment who showed very low levels of miR-21 (0.01) before operation, whereas high levels (17.78) were detected after the occurrence of metastasis (Table 4). Further studies are needed to clarify whether the occurrence of metastasis after operation leads to a normal expression range of miR-21 or abnormally high expression is due to metastasis.

The incidence of BC in women aged 65 years and older has increased from 25.3% to 32.9% in the last 10 years^2^. The risks of BC in Japanese women is thought to have increased because of the consumption of Western-style foods, increased obesity, early age at menarche, late age at first pregnancy, late age at menopause, and an increased aging population^2,42–44^. BC screening is important in both Japan and worldwide.

## Methods

### Study population

This study was conducted at Sanno Hospital (Minato-ku, Tokyo, Japan), the Kitasato University Medical Center (Kitamoto City, Saitama, Japan), and the International University of Health and Welfare Hospital (Nasu-Shiobara City, Tochigi Japan) between July 1, 2016 and November 30, 2018. Prior to implementation, the study was approved by the ethics committee of the three institutions (#16-S-8 Sanno Hospital under the authority of IUHW on June 26, 2016 and on March 15, 2017; #28–51 Kitasato University Medical Center on March 27, 2017; #13-B-287 IUHW Hospital on March 22, 2018). All patients provided informed consent by formal letter for this study. Twenty-two patients with BC were diagnosed by mammography, echography, MRI, and subsequent needle biopsy before surgery, and the final pathological diagnosis was delivered by surgical resection at Sanno Hospital (Table 1 and Supplementary Table 1). Urine samples were collected before surgical treatment before breakfast as the first morning urine and stored at −80 °C until the exosomes were separated. All urine samples were collected before the patients had been administered neo-adjuvant chemotherapy or showed evident distant metastasis.

The control urine samples were collected from 26 healthy females who visited the Health Care Center, the International University of Health and Welfare Hospital for a health check. No subjects had complaints, abnormal physical findings including obesity and blood hypertension, abnormal peripheral blood examination and blood chemistry, or abnormal examinations of ECG, abdominal echography, upper GI endoscopic findings, breast mammography, and echography. They also had no history of cancer, signs of dysplasia, inflammatory disease, autoimmune disease, or chronic diseases such as cardiac, liver, or kidney diseases (Supplementary Table 2).

### Classification of breast cancer clinical stage

The clinical stage was classified according to the American Joint Committee on Cancer tumour-lymph node-metastasis (TNM) classification system^45^ as shown in Table 1. The 22 patients with BC showed no metastasis and were in the very early stage of BC.

Classification of Luminal A, Luminal B, Her2, and TN for selecting BC treatment was performed to evaluate sensitivity and specificity^46^ (Supplementary Table 1).

### Isolation of urinary exosomes

All samples were transferred to Kitasato University Medical Center at −80 °C and urinary exosomes were isolated to analyse the expression of miR-21 by RT-PCR and the expression of MMP-1/CD63 by western blotting. Urinary exosomes were isolated with an Exosome Isolation Kit (Miltenyi Biotec) and were confirmed to be exosomes by western blotting with biotin-conjugated anti-CD63 antibody (BioLegend, San Diego, CA, USA). The isolated exosomes were also identified by electron microscopy^15^. Exosomes were layered on a carbon/Formvar film-coated TEM grid (Okenshoji Co., Ltd., Tokyo Japan) for 10 min. They were stained with 2% uranyl acetate for 2 min. Observation was performed with a transmission electron microscope (Hitachi H-7600, Tokyo, Japan).

A volume of 2 mL urine was used from all samples collected from patients with BC and healthy controls for exosome isolation. Before exosome isolation, the thawed urine was centrifuged at 3,000 × *g* for 15 min at 4 °C and passed through 0.22-μm nylons filter. The RNA was extracted from isolated exosomes using a Total Exosome RNA & Protein Isolation Kit (Thermo Fisher Scientific, Waltham, MA, USA), and cDNA was synthesized from the acquired microRNA template with a reverse transcription kit (TaqMan MicroRNA Reverse Transcription Kit, Thermo Fisher Scientific). We performed miRNA extraction after quantifying the amount of urine exosomes using a PS Capture Exosome ELISA Kit (Wako Pure Chemical Industries, Osaka, Japan). Furthermore, the miRNA amount between numerous samples was corrected by performing a reverse transcription reaction using 5 ng of the collected miRNA for expression analysis.

### Analysis of miR-21 extracted from urinary exosomes

Quantitative RT-PCR was carried out in a Step One Plus^®^ (Applied Biosystems, Foster City, CA, USA) for miR extracted from urinary exosomes. An miR-21 assay (Applied Biosystems) was performed for miR expression assays according to the manufacturer’s instructions. Assays were performed in duplicate tubes for each sample.

### Comparison of miR-21 expression levels in urine exosomes

Based on the mean number of miR-21 copies in the 26 healthy controls, the copy number of miR-21 in patients with BC was calculated as the relative expression grade. ΔCT was determined by subtracting the mean CT value of miR-21 in healthy controls from the individual CT value of miR-21 in patients with BC. Finally, the copy number of miR-21 was compared as the value of 2^ΔCT^. Similarly, the value was compared to preoperative and postoperative expression in 7 patients with BC.

### Comparison of MMP-1/CD 63 expression levels in urinary exosomes

The expression levels of MMP-1 and CD63 were determined by western blotting. Expression of MMP-1/CD63 was examined using an anti-MMP-1 antibody (abcam plc., Cambridge, UK) and biotin-conjugated anti-CD63 antibody (BioLegend). Their expression were pixeled and the ratio of MMP-1/CD63 in the 22 patients with BC was compared to those in the 26 controls with Image J 1.52a software^47^. Similarly, preoperative and postoperative expression was compared in 7 patients with BC, which revealed identical results. To validate reproducibility, all experiments were performed twice, and the average or median value was confirmed.

### Statistical analysis

All values are expressed as the mean ± standard deviation (SD) or median [95% confidence interval (CI)]. Statistical analysis was performed using PRISM 7 for Mac OS X (GraphPad Software, Inc., La Jolla, CA, USA). Data were tested for normality and equal variance to confirm the appropriateness of parametric tests. Data followed a normal distribution were analysed by Student *t* test. Mann-Whitney *U* tests were used to evaluate differences between groups: a *p* value < 0.05 was regarded as statistically significant.

### Ethics approval

All procedures performed in this study were in accordance with the ethical standard of the institutional and national research committee and with the 1964 Helsinki declaration and its later amendments or comparable ethical standards. The study was approved by the ethics committees of the three institutions (#16-S-8 Sanno Hospital under the authority of IUHW on June 26, 2016 and on March 15, 2017; #28–51 Kitasato University Medical Center on March 27, 2017; #13-B-287 IUHW Hospital on March 22, 2018). This article does not contain any studies with animals performed by any of the authors.

## Conclusions

Measuring the expression of both miR-21 and MMP-1/CD63 in urine exosomes is an effective, reliable, inexpensive, and simple screening test. Further studies are needed to confirm these results in a larger sample size.

## Supplementary information

Supplementary material

## Data Availability

All data generated or analysed during this study are included in this published article.
