# Supplementary material for: Novel breast cancer screening: combined expression of miR-21 and MMP-1 in urinary exosomes detects 95% of breast cancer without metastasis
Source: Sci Rep. 2019 Sep 19;9:13595. doi: 10.1038/s41598-019-50084-5 (PMC6753125; doi:10.1038/s41598-019-50084-5)
Supplement: Supplementary file 1 — Supplementary material [file 41598_2019_50084_MOESM1_ESM.pdf]

Novel breast cancer screening: combined expression of miR-21 and MMP-1 in urinary exosomes detects 95% of breast cancer without metastasis

Wataru Ando [1], Kiyoshi Kikuchi [2], Takayuki Uematsu [3], Hiroaki Yokomori [4], Takashi Takaki [5], Masaya Sogabe [6], Yutaka Kohgo [7, 8], Katsuya Otori [1], Shigemi Ishikawa [9], Isao Okazaki [7, 8, 10]

[1] Department of Clinical Pharmacy, Center for Clinical Pharmacy and Sciences, Kitasato University School of Pharmacy, 5-9-1 Shirokane, Minato-ku, Tokyo 108-8641, Japan, Tel +81-3440-3055

[2] Department of Surgery, Sanno Hospital, International University of Health and Welfare, 8-10-16 Akasaka, Minato-ku, Tokyo 107-0052, Japan, Tel +81-3-3402-3151

[3] Biomedical Laboratory, Division of Biomedical Research, Kitasato University Medical Center, 6-100 Arai, Kitamoto City, Saitama 364-8501, Japan, Tel +81-48-593-1212

[4] Department of Internal Medicine, Kitasato University Medical Center, 6-100 Arai, Kitamoto City, Saitama 364-8501, Japan, Tel +81-48-593-1212

[5] Division of Electron microscopy, Showa University School of Medicine, 1-5-8 Hatanodai, Shinagawa-ku, Tokyo 142-8555, Japan Tel +81-3-3784-8056

[6] Department of General Thoracic Surgery, Jichi Medical University, 3311-1 Yakushiji, Shimotsuke, Tochigi 329-0498, Japan, Tel +81-285-44-2111

[7] Department of Internal Medicine, International University of Health and Welfare Hospital, 537-3 Iguchi, Nasu-Shiobara, Tochigi 329-2763, Japan, Tel +81-287-39-3060

[8] Health Care Center, International University of Health and Welfare Hospital, 537-3 Iguchi, Nasu-Shiobara, Tochigi 329-2763, Japan, Tel +81-287-39-3060

[9] Department of Chest Surgery, International University of Health and Welfare Hospital, 537-3 Iguchi, Nasu-Shiobara, Tochigi 329-2763, Japan, +81-287-39-3060

[10] Department of Internal Medicine, Sanno Hospital, International University of Health and Welfare, 8-10-16 Akasaka, Minato-ku, Tokyo 107-0052, Japan, Tel +81-3-3402-3151

Supplementary Materials

Supplementary Table 1. Breast Cancer Cases, Characteristics of Pathology and Clinical Data

No.	ER (%)	PR (%)	Her2	CEA (ng/ml)	CA 15-3 (U/ml)	CRNN (mg/dl)	Alcohol (g/day)	Tabaco (cigarettes /day)
1	<1	<1	0	1.2	16.9	0.69	—	—
2	0	0	3	1.1	10.6	0.76	—	—
3	>90	90	1+	0.7	7.4	0.66	20	—
4	>90	90	0	1.6	5.7	0.50	—	—
5	0	0	0	1.6	26	0.53	—	—
6	<1	<1	3+	2.6	16.8	0.60	—	—
7	>90	>90	0	1.9	18.8	0.66	—	—
8	80-90	70-80	0	1.2	9.5	0.75	—	—
9	>90	>90	1+	1.6	7.9	0.60	40 g/week	—
10	>90	>90	1+	4.8	26	0.81	—	—
11	>80	>90	1+	1.3	8.8	0.62	20	—
12	>90	>90	1+	1.2	6.4	0.68	43	—
13	50	5	2+	2.6	10.6	0.50	—	—
14	0	0	0	2	9.1	0.66	—	—
15	50	5	2+	1	7.5	0.52	16	—
16	>90	>90	1+	1.8	11.2	0.75	20	—
17	0	0	0	3	16.3	0.70	20	—
18	>90	>90	0	3.4	5.5	0.45	—	—
19	80	50	1+	1.7	46.1	0.41	—	—
20	>90	>90	1+	2.3	8.3	0.54	20	—
21	>90	>90	0	1.4	19.7	0.60	—	—
22	80	30	0	1.2	16.9	0.58	—	—

ER, Oestrogen receptor; PR, progesterone receptor; Her2, human epidermal growth factor receptor 2; CEA, Carcinoembryonic antigen; CA 15-3, carbohydrate antigen 15-3; CRNN, serum creatinine;

Supplementary Table 2. The Characteristics of Healthy Controls

No.	Age (years)	Body Weight (kg)	BMI (kgm^2)	Expression Levels of miR-21 ($2^{\Delta Ct}$)*	Expression Levels of MMP-1/CD63**	CRNN (mg/dl)	Alcohol (g/day)	Tabaco (cigarettes /day)
1	34	50.0	20.6	1.34	0.40	0.71	-	-
2	36	58.0	21.7	0.68	1.31	0.56	-	-
3	33	47.5	18.7	0.97	0.20	0.59	20	-
4	35	43.0	21.1	0.18	0.30	0.6	-	-
5	38	54.3	21.8	0.50	0.14	0.72	Sometime 20	-
6	45	54.1	22.1	0.35	0.15	0.54	-	-
7	49	50.0	17.9	1.69	0.56	0.51	-	-
8	47	52.1	21.4	0.46	0.86	0.56	-	-
9	48	68.8	23.2	0.29	0.71	0.67	20	-
10	44	44.6	19.2	0.15	0.90	0.51	-	-
11	44	68.0	25.2	0.03	18.31	0.51	-	-
12	45	52.4	23.3	1.59	2.86	0.46	-	11~20
13	41	62.7	24.5	7.17	2.24	0.54	-	-
14	45	54.6	22.1	4.75	0.5	0.76	43	-
15	40	59.4	16.6	3.29	0.5	0.63	Sometime 20	-
16	42	65.8	24.6	6.65	0.56	0.84	-	-
17	49	58.2	24.3	0.27	0.51	0.56	60	20
18	55	48.9	20.6	0.63	0.91	0.66	-	-
19	56	49.1	19.3	0.45	0.96	0.62	20	-
20	54	59.3	22.8	1.03	0.43	0.75	43	No, but past 10 years
21	52	58.2	24.3	1.41	0.42	0.56	30	No, but past 5 years
22	50	53.6	21.7	8.78	0.29	0.69	43	-
23	69	50.2	20.2	1.69	0.35	0.66	-	-
24	71	49.8	21.2	0.09	0.78	0.99	-	-
25	71	61.6	25.2	10.60	0.36	0.62	Sometime 20	-
26	73	54.6	23.1	1.98	0.61	0.68	-	-

*number indicates the relative expression levels, that is microRNA copies by RT-PCR in patients divided

the mean number of microRNA copies in 26 healthy controls.

**number indicates the relative expression levels of MMP-1/CD63, that was measured by western blotting by both antibodies.

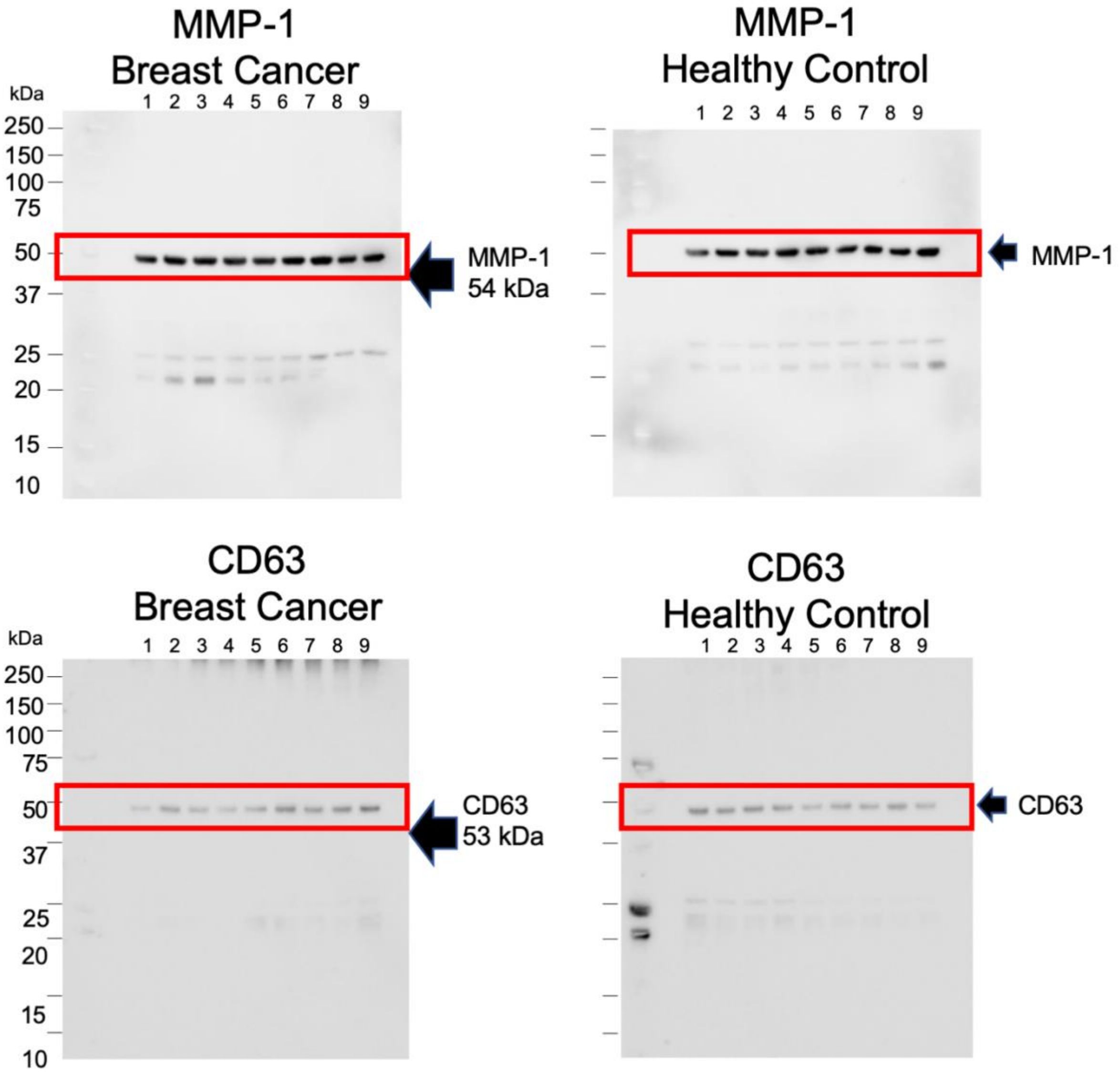

Supplementary Figure 1. The full-length blot of western blotting analysis in the protein from exosome with anti-MMP-1 and anti-CD63 antibody. The red-enclosed section was used for Figure 3.
